# A Pyridine-Containing Cu^2+^-Selective Probe Based on Naphthalimide Derivative

**DOI:** 10.3390/s141224146

**Published:** 2014-12-15

**Authors:** Jun Zhang, Qiang Wu, Bangliang Yu, Chunwei Yu

**Affiliations:** 1 Laboratory of Environmental Monitoring, School of Tropical and Laboratory Medicine, Hainan Medical University, Haikou 571101, China; E-Mail: jun_zh1979@163.com; 2 Faculty of Laboratory Medicine, School of Tropical and Laboratory Medicine, Hainan Medical University, Haikou 571101, China; E-Mail: wuqiang001001@aliyun.com; 3 Department of Pharmacy, Hainan Medical University, Haikou 571101, China; E-Mail: yubangliang@126.com

**Keywords:** fluorescent probe, naphthalimide, Cu^2+^, schiff base, pyridine

## Abstract

A new fluorescent probe **P** derived from naphthalimide bearing a pyridine group has been synthesized and characterized. The proposed probe **P** shows high selectivity and sensitivity to Cu^2+^ in aqueous media. Under optimized conditions, the linear response of **P** (2 μM) toward Cu^2+^ was 0.05–0.9 μM in ethanol-water solution (3:2, v:v, 50 mM HEPES, pH 7.4), and the detection limit was 0.03 μM.

## 1. Introduction

Development of optical sensors for the detection of environmental targets has been an actively research topic recently. Because of its simplicity and high sensitivity, the fluorescence technique has become a powerful tool among the methods available for chemical sensors [1]. Most metal ions play important roles in living systems and have an extreme eco-toxicological impact on the environment and humans. Among these various metal ions, Cu^2+^ is both a significant environment pollutant and an essential trace element in biological systems [2], so detecting the presence of Cu^2+^ has received considerable attention. So far, many Cu^2+^-selective fluorescent probes have been successfully devised [2–24]. It is unfortunate that only a few examples of “off-on” type probes are available due to the fluorescence quenching nature of paramagnetic Cu^2+^ [2–20]. In most practical applications, fluorescence quenching changes in fluorescence intensity can be interrupted by many other poorly quantified or variable factors such as photobleaching, probe molecule concentration, the environment around the probe molecule (pH, polarity, temperature, and so on), and stability under illumination, *etc.* [21–23]. To increase the selectivity and sensitivity of a measurement for analytical purposes, probes in which the binding of Cu^2+^ leads to a fluorescence enhancement are desirable. Therefore, there is still room to develop highly sensitive and selective “off-on” probes for Cu^2+^ in neutral aqueous media.

**Scheme 1.**
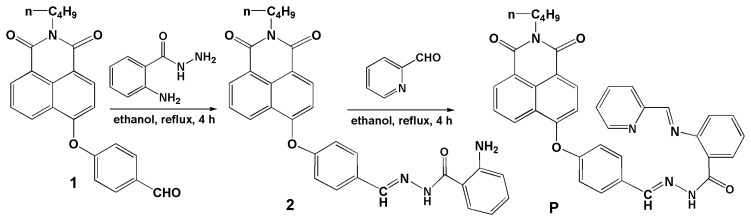
The synthesis route of compound **P**.

For the construction of a highly efficient probe for a target, it is necessary to choose an efficient fluorophore and consider the geometry of the coordination sites of the target [2,3,23]. Naphthalimide derivatives, which are widely used as fluorescent dyes, have excellent photophysical properties, such as high fluorescence quantum yields, large Stokes shifts, strong absorption band and stability. Furthermore, the recognition moiety should be preliminarily considered in designing probes because they are responsible for the selectivity and binding efficiency of the whole probe. According to Hard-Soft-Acid-Base theory, O and N donor atoms established the high affinity for Cu^2+^ [14]. With this intention, a Cu^2+^-specific “off-on” type fluorescent probe **P** derived from naphthalimide with N and O as coordination sites was designed and synthesized (Scheme 1).

## 2. Experimental Section

### 2.1. Reagents and Instruments

All of the materials were analytical reagent grade and used without further purification. The metal ions and anions salts employed are NaCl, MgCl_2_·6H_2_O, CdCl_2_, HgCl_2_, CaCl_2_·2H_2_O, FeCl_3_·6H_2_O, CrCl_3_·6H_2_O, Zn(NO_3_)_2_·6H_2_O, AgNO_3_, CoCl_2_·6H_2_O, MnCl_2_·4H_2_O, CuCl_2_·2H_2_O, NiCl_2_·6H_2_O, PbCl_2_, NaClO, NaNO_3_, Na_2_CO_3_, NaCl, NaAc, NaClO_4_, KBr and Na_2_HPO_4_, respectively. NMR spectra were recorded in DMSO-*d*_6_ at 25 °C on a Bruker WM-300 spectrometer (Fällanden, Switzerland). Electrospray ionization (ESI) analyses were performed on a Thermo TSQ Quantum Mass Spectrometer (Waltham, MA, USA). UV-Vis spectra were obtained on a Beckman DU-800 spectrophotometer (Bremen, Germany) with 1 cm quartz cell at 25 °C. Fluorescence measurements were carried out on a HORIBA Fluoromax-4 luminescence spectrometer (Paris, France). Fluorescence imaging was performed by confocal fluorescence microscopy on an Olympus FluoView Fv1000 laser scanning microscope (Osaka, Japan). pH values were measured with a PBS-3C pH-meter (Shanghai, China).

### 2.2. Synthesis

Compounds **1** and **2** were obtained according to our previous work [25]. Briefly, under N_2_, compound **1** (373.1 mg, 1.0 mmol) and anthraniloyl hydrazine (181.3 mg, 1.2 mmol) were combined in ethanol (50 mL). The reaction solution was refluxed for 4 h and stirred. The precipitate so obtained was filtered and washed three times with ethanol. The crude product was purified by recrystallization from ethanol to give light yellow crystals of **2**. Yield: 75%. MS: *m/z* 507.10 [M + H]^+^. ^1^H-NMR (DMSO-*d*_6_, δ ppm): 11.65 (s, 1 H), 8.64 (d, 1 H, *J* = 8.35), 8.55 (d, 1 H, *J* = 8.15), 8.43 (t, 2 H, *J* = 7.42), 7.90 (t, 1 H, *J* = 7.82), 7.85 (d, 1 H, *J* = 8.20), 7.58 (d, 1 H, *J* = 7.85), 7.36 (d, 2 H, *J* = 8.35), 7.21 (t, 1 H, *J* = 7.65), 7.13 (d, 1 H, *J* = 8.25), 6.77 (d, 1 H, *J* = 8.30), 6.59 (t, 1 H, *J* = 7.47), 6.40 (b, 2 H), 4.04 (t, 2 H, *J* = 7.32), 1.62 (m, 2 H, *J* = 7.41), 1.36 (m, 2 H, *J* = 7.37), 0.93 (t, 3 H, *J* = 7.35). ^13^C-NMR (DMSO-*d*_6_, δ ppm): 163.86, 163.22 (C=O), 158.71, 156.54, 150.54, 133.07, 132.73, 132.21, 131.96, 129.59, 129.44, 128.60, 127.72, 123.96, 122.68, 121.04, 117.22, 116.85, 115.06, 112.63, 36.25, 30.14, 20.27, 14.19.

Compound **P**: Compound **2** (506.2 mg, 1.0 mmol) and 2-pyridinecarboxaldehyde (128 μL, 1.2 mM) were reacted in refluxing ethanol (50 mL) for 4 h, and then cooled to room temperature, the precipitate so obtained was purified by recrystallization from ethanol to give light yellow crystals of **P**. Yield: 72.5%. MS: *m/z* 596.15 [M + H]^+^. ^1^H-NMR (DMSO-*d*_6_, δ ppm): 8.98 (s, 1 H), 8.62 (d, 1 H, *J* = 8.35), 8.55 (d, 1 H, *J* = 7.25), 8.52 (d, 1 H, *J* = 7.25), 8.20 (d, 1 H, *J* = 8.25), 7.91 (s, 1 H), 7.88 (d, 1 H, *J* = 7.85), 7.84 (s, 1 H), 7.83 (s, 1 H), 7.80 (d, 1 H, *J* = 9.50), 7.73 (d, 1 H, *J* = 9.20), 7.43 (d, 1 H, *J* = 7.95), 7.34 (s, 1 H), 7.32 (s, 2 H), 7.27 (t, 1 H, *J* = 8.45), 7.10 (d, 1 H, *J* = 8.25), 6.79 (d, 1 H, *J* = 8.00), 6.73 (t, 1 H, *J* = 7.92), 6.49 (d, 1 H, *J* = 3.30), 4.03 (t, 2 H, *J* = 7.40), 1.61 (m, 2 H, *J* = 7.46), 1.35 (m, 2 H, *J* = 7.43), 0.92 (t, 3 H, *J* = 7.35). ^13^C*-*NMR (DMSO-*d*_6_, δ ppm): 163.86, 163.21, 161.25 (C=O), 158.78, 158.72, 156.89 (ArC), 150.03, 149.68 (C=N), 146.30, 137.57, 134.30, 133.03, 132.28, 131.95, 129.96, 129.42, 128.58, 128.46, 127.70, 124.04, 123.90, 122.67, 121.31, 121.07, 118.22, 117.20, 115.49, 115.15, 112.58, 30.14, 20.26, 14.18 (see Supplementary Material, Figures S1–S3).

### 2.3. General Procedure for Spectroscopic Measurements

A stock solution of **P** (1 mM) was prepared in DMSO. To 5 mL glass tubes, **P** (10 μL, 1 mmol) and a proper amount of Cu^2+^ stock solution (1.0 mmol) were added succesively and then diluted with ethanol-water solution (3:2, v:v, 50 mM HEPES, pH 7.4). The resulting solution was thoroughly mixed. For all measurements, excitation and emission slit widths were 2 nm, excitation wavelength was 360 nm.

## 3. Results and Discussion

### 3.1. pH Effects on **P** and **P** with Cu^2+^

**Figure 1.**
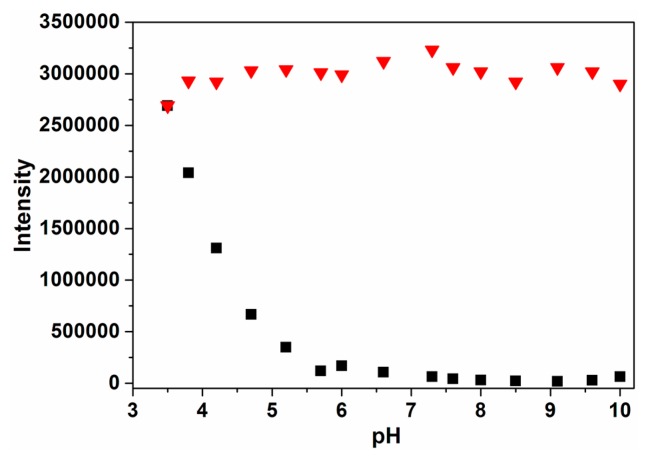
Influence of pH on the fluorescence spetra of **P** (2 μM, ■) and **P** (2 μM, ▼) plus Cu^2+^ (50 μM) in ethanol-water solution (3:2, v:v). The pH was modulated by adding 1 M HCl or 1 M NaOH in HEPES buffers.

The influence of pH on the fluorescence response of probe **P** was determined first (Figure 1). At pH below 5.7, the fluorescence response of **P** was affected by pH to some extent. With the increase of pH from 5.7 and 10.0, “off-on” fluorescence signals at 432 nm were mainly caused by the addition of Cu^2+^. This indicated that the receptor gradually captured Cu^2+^ and formed the **P**-Cu^2+^ complex. In this work, pH 7.4 was chosen as an optimum experimental condition in that **P** could work with very low background fluorescence.

### 3.2. Fluorescence Spectral Response of **P**

**Figure 2.**
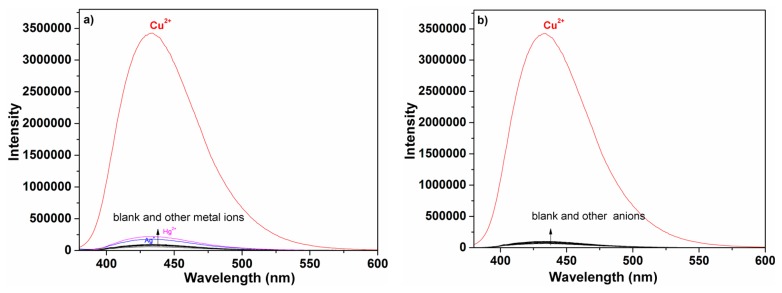
(**a**) Fluorescence spectra of **P** (2 μM) with different metal ions or (**b**) anions (50 μM) in ethanol-water solution (3:2, v:v, 50 mM HEPES, pH 7.4).

**Figure 3.**
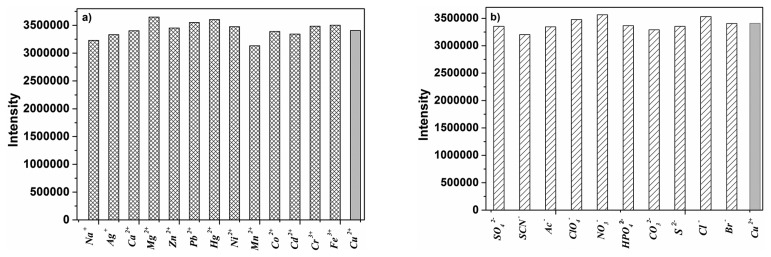
(**a**) Fluorescence response of **P** (2 μM) to 10 μM of Cu^2+^ or to the mixture of 50 μM individual metal ions with 10 μM of Cu^2+^ in ethanol-water solution (3:2, v:v, 50 mM HEPES, pH 7.4); (**b**) Fluorescence response of **P** (2 μM) to 10 μM of Cu^2+^ or to the mixture of 50 μM individual anions with 10 μM of Cu^2+^.

An important feature of **P** was its selectivity toward Cu^2+^ over other competitive species, and the selectivity experiments for probe **P** were conducted as shown in Figure 2. Fluorescence spectral changes of **P** were examined with addition of metal ions and anions including Na^+^, K^+^, Ag^+^, Mg^2+^, Ca^2+^, Zn^2+^, Pb^2+^, Cd^2+^, Co^2+^, Ni^2+^, Mn^2+^, Hg^2+^, Cu^2+^, Cr^3+^, Fe^3+^, Al^3+^, S^2−^, SO_4_^2−^, SCN^−^, NO_3_^−^, CO_3_^2−^, Cl^−^, Ac^−^, ClO_4_^−^, Br^−^ and HPO_4_^2−^. An obvious enhancement of fluorescence intensity at 432 nm was observed only upon addition of Cu^2+^, which was attributable to the complexation between **P** and Cu^2+^. In contrast, no obvious changes were observed in the case of other metal ions and anions. Moreover, to check the interferences from other metal ions and anions on the fluorescence signal of Cu^2+^, competition experiments were performed between Cu^2+^ and selected metal ions and anions (Figure 3). When selected metal ions and anions (50 μM) were added into ethanol-water solution (3:2, v:v, 50 mM HEPES, pH 7.4) of **P** (2 μM) containing Cu^2+^ (10 μM), the emission spectra displayed a similar pattern to that with Cu^2+^ alone. This experiment clearly demonstrated that selected metal ions and anions even in higher concentrations did not interfere the Cu^2+^ detection, which made it applicable for Cu^2+^ sensing in the real sample.

**Figure 4.**
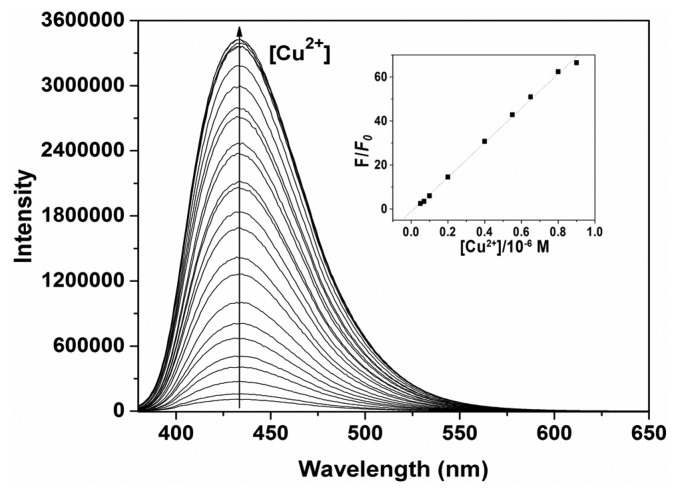
Fluorescence spectra of **P** (2 μM) in ethanol-water solution (3:2, v:v, 50 mM HEPES, pH 7.4) in the presence of different amounts of Cu^2+^. Inset: Fluorescence intensity at 432 nm as a function of Cu^2+^ concentration.

In addition, the titration of **P** with various amounts of Cu^2+^ in ethanol-water solution (3:2, v:v, 50 mM HEPES, pH 7.4) were studied (Figure 4).

As the Cu^2+^ concentration increased, the fluorescence emission intensity at 432 nm gradually increased accordingly. The linear fluorescence enhancement of **P** (2 μM) to Cu^2+^ was obtained in the range of 0.05–0.9 μM (*R* = 0.999; inset of Figure 4). The limit of detection (LOD) was 0.03 μM, based on 3 × δ_blank_/*k* (where δ_blank_ is the standard deviation of the blank solution and *k* is the slope of the calibration plot).

### 3.3. The Binding Mechanism

**Figure 5.**
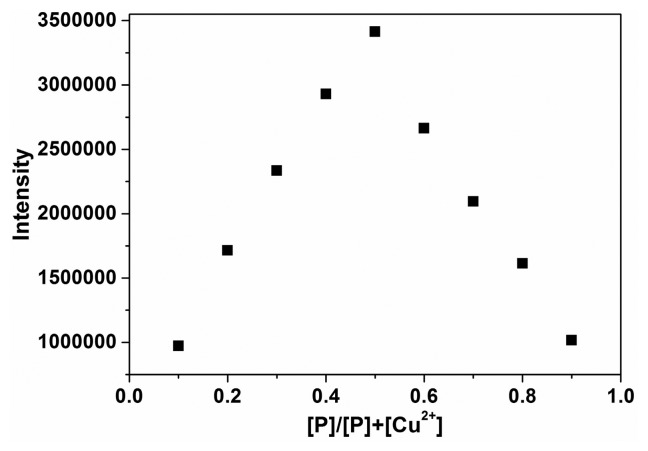
Job's plot for **P-**Cu^2+^ complex, keeping the total concentration of **P** and Cu^2+^ as 10 μM.

To quantify the complexation ration between **P** and Cu^2+^, a Job plot experiment was carried out by keeping the total concentration of **P** and Cu^2+^ at 10 μM (Figure 5). The results suggested that a 1:1 complex of **P** with Cu^2+^ was formed, which was supported by the presence of a peak at *m/z* 659.2 corresponding to **P**-Cu^2+^ in the ESI-MS spectrum of the components of the mixture of **P** and 1 equivalent Cu^2+^ in ethanol (Supplementary Material, Figure S4). The ^1^H-NMR spectra also indicated the binding of **P** with Cu^2+^ (Supplementary Material, Figures S5 and S6). The association constant K was determined from the slope to be 6.2 × 10^5^ M^−1^, by plotting the fluorescence intensity 1/(F − F_0_) against 1/[Cu^2+^].

**Figure 6.**
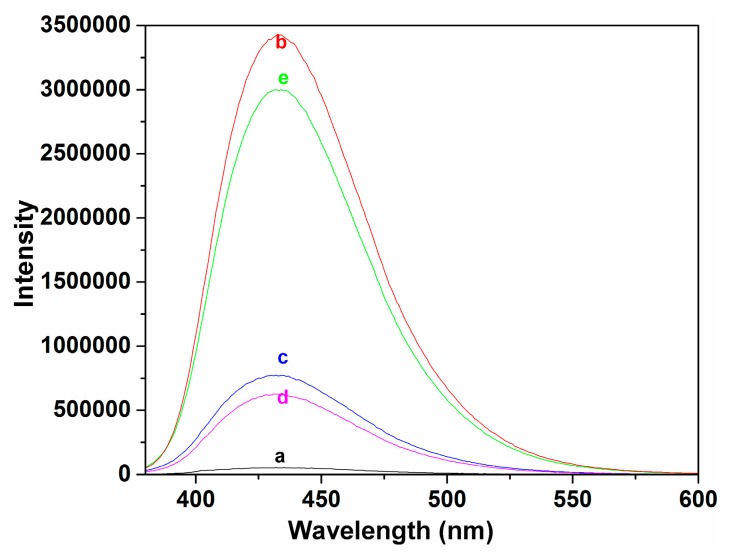
Fluorescence spectra in ethanol-water solution (3:2, v:v, 50 mM HEPES, pH 7.4). a: **P** (2 μM); b: **P** (2 μM) + Cu^2+^ (50 μM); c: **P** (2 μM) + Cu^2+^ (50 μM) + EDTA (100 μM); d: **P** (2 μM) + Cu^2+^ (50 μM) + EDTA (100 μM) + Cu^2+^ (100 μM); e: **P** (2 μM) + Cu^2+^ (50 μM) + EDTA (200 μM) + Cu^2+^ (100 μM).

**Scheme 2.**
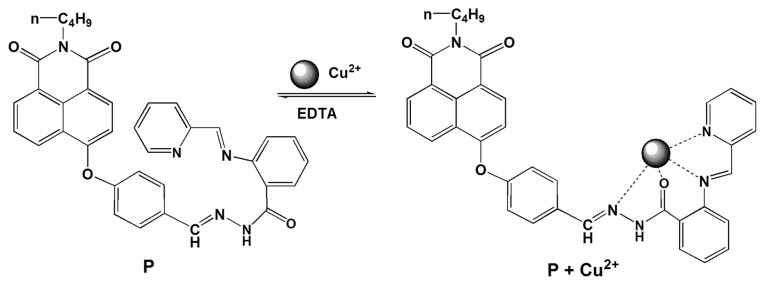
The mode of formation of **P-**Cu^2+^ complex.

From Figure 2, no significant changes in fluorescence spectra were observed when probe **P** was exposed to other metal ions and anions. We believe that this is due to a rapid isomerization of the C=N double bond in the excited state [26,27], though other mechanisms such as photoinduced electron transfer (PET) may also contribute to it. Notably, by adding Cu^2+^, the fluorescence character of **P** was different from free **P** and other metal ions and anions, its fluorescence at λ_em_ 432 nm was turned from “off” to “on”. The enhancement of fluorescence was likely due to restriction of acyclic C=N isomerization in the Schiff base upon addition of Cu^2+^ [25–27]. Accordingly, the proposed binding mode of **P** with Cu^2+^ can be illustrated as in Scheme 2. It is believed that this process is reversible, which has been proved by a test using EDTA-Cu^2+^ (Figure 6). As seen, in absence of Cu^2+^, probe **P** had a weak fluorescence. Addition of Cu^2+^ led to a reversible coordination with the ligand, resulting in an appearance of fluorescence enhancement at λ_em_ 432 nm. Thus, an “off-on” based fluorescent probe for Cu^2+^ was implemented.

## 4. Conclusions

In summary, an efficient “off-on” probe **P** for Cu^2+^ was proposed. Our studies showed that **P** was a highly selective and sensitive probe for Cu^2+^, which could work in neutral aqueous solution media and has great potential use in environmental sensing applications. These results open up new possibilities for the construction of “off-on” probes for other metal ions.
